# Racial and Ethnic Diversity in Clinical Trials for Disease Modifying Drugs in Parkinson Disease: A Systematic Review & Meta-Analysis

**DOI:** 10.1002/mdc3.70482

**Published:** 2025-12-30

**Authors:** Farley Reis Rodrigues, Gabriel Bolner, Giovana Barros e Silva Ribeiro, Aishwarya Koppanatham, Raul Vinícius da Silva, Conor Fearon, Artur F. Schumacher Schuh, Ece Bayram, Juan Blas M. Couto, Mwiza Ushe, Daniel G. Di Luca

**Affiliations:** 1 Federal University of Vicosa, Viçosa, Brazil; 2 Federal University of Health Sciences of Porto Alegre, Porto Alegre, Brazil; 3 State University of Campinas, Campinas, Brazil; 4 Andhra Medical College, Visakhapatnam, India; 5 Federal University of Santa Catarina, Florianópolis, Brazil; 6 Dublin Neurological Institute at the Mater Misericordiae University Hospital, Dublin, Ireland; 7 Federal University of Rio Grande do Sul, Porto Alegre, Brazil; 8 Hospital de Clínicas de Porto Alegre, Porto Alegre, Brazil; 9 University of Colorado Anschutz, Aurora, Colorado, USA; 10 Cognitive and Translational Institute of Neurosciences, Buenos Aires, Argentina; 11 Washington University in St. Louis, St. Louis, Missouri, USA

**Keywords:** disease-modifying therapies, epidemiology, Parkinson’s disease, pharmacology, representativeness

## Abstract

**Background:**

There is a historic underrepresentation of non-White participants in Parkinson’s Disease (PD) research, although this has not been explored in trials for potential disease-modifying therapies.

**Objective:**

To evaluate the representation of racial/ethnic minority patients enrolled in double-blind, randomized, placebo-controlled clinical trials (DBRCTs) for PD.

**Methods:**

A systematic search of four electronic databases was performed. DBRCTs evaluating pharmacological therapies for disease modification in PD were included. Data extraction followed PRISMA guidelines. We computed demographic data with pooled prevalence and 95% confidence intervals (CIs).

**Results:**

Among 37 DBRCTs and 11,022 patients, 19 studies (51.4%) reported race/ethnicity, being 1.4% Asian, 0.19% Black, and 0.17% Hispanic. Pooled prevalence of participants identified as White in clinical trials was 98% (CI 0.97–0.99, *P* < 0.001).

**Conclusion:**

Racial and ethnic minorities were disproportionately underrepresented in DBRCTs for potential disease-modifying therapies for PD. Additional efforts are required to increase the racial and ethnic representation in such studies.

The global prevalence of PD has been estimated at 11.8 million cases, reflecting a 273.9% increase from 1990 to 2021.^1–3^ Notably, low- and middle-income countries account for nearly 85% of disability-adjusted life-years (DALYs) attributable to PD, underscoring its disproportionate burden in resource-limited settings.^2,4^

Despite this global distribution, racial and ethnic minorities with PD have been historically excluded from clinical research, potentially limiting the generalizability of results.^5^ This applies to genetic and epidemiological studies, although little is known about the inclusion of underrepresented groups in diseasemodifying clinical trials. Considering the lack of effective therapies to date, it is pivotal to ensure the inclusion of a diverse population to determine effectiveness across all racial and ethnic groups.

In this study, we conducted a systematic review and meta-analysis to understand the racial and ethnic group representation in disease-modifying clinical trials for PD.

## Methods

This systematic review and meta-analysis were performed and reported in accordance with the Cochrane Collaboration Handbook for Systematic Review of Interventions and the Preferred Reporting Items for Systematic Review and Meta-Analysis (PRISMA) Statement guidelines.^6,7^ The prospective meta-analysis protocol was registered on PROSPERO on 20 October 20, 2024, under protocol CRD42024599403.

### Study Eligibility

To allow comparison between studies, inclusion was restricted to studies that met all the following eligibility criteria: (1) randomized double-blind clinical trials (RCTs); (2) studies evaluating pharmacologic potential disease-modifying treatments for PD. We excluded: (1) non-randomized studies; (2) studies on drugs not used as potential modifiers of the clinical course of PD; (3) studies not reported in English; and (4) non-double-blind clinical trials.

### Search Strategy and Data Extraction

We systematically searched PubMed, Embase, Cochrane Central Register of Controlled Trials, and clinicaltrials.gov from inception to September 2024. The complete search strategy used can be accessed in the Appendix (Supplementary File S1). Three authors (F.R., R.V., and G.B.) independently screened the studies based on title and abstract, following predefined search criteria. A fourth author (D.G.D.L.) solved the conflicts. Two authors (F.R. and G.B.) extracted the data and performed the quality assessment (F.R. and A.K.).

### Endpoints and Subgroup Analysis

The primary outcome of interest was the proportion of under-represented minorities in clinical trials. Definitions of ethnicity and race varied in clinical trials, although they were predominantly self-reported.

### Statistical Analysis

*P* values and confidence intervals (CIs) reflect the pooled prevalence estimates of White participants across studies. The statistical significance relates to the aggregated prevalence differences. R statistical software, version 4.4.0 (R Foundation for Statistical Computing) was used for statistical analysis, and Python, version 3.10.12 was used for generating images.

## Results

### Study Characteristics

**Figure 1.**
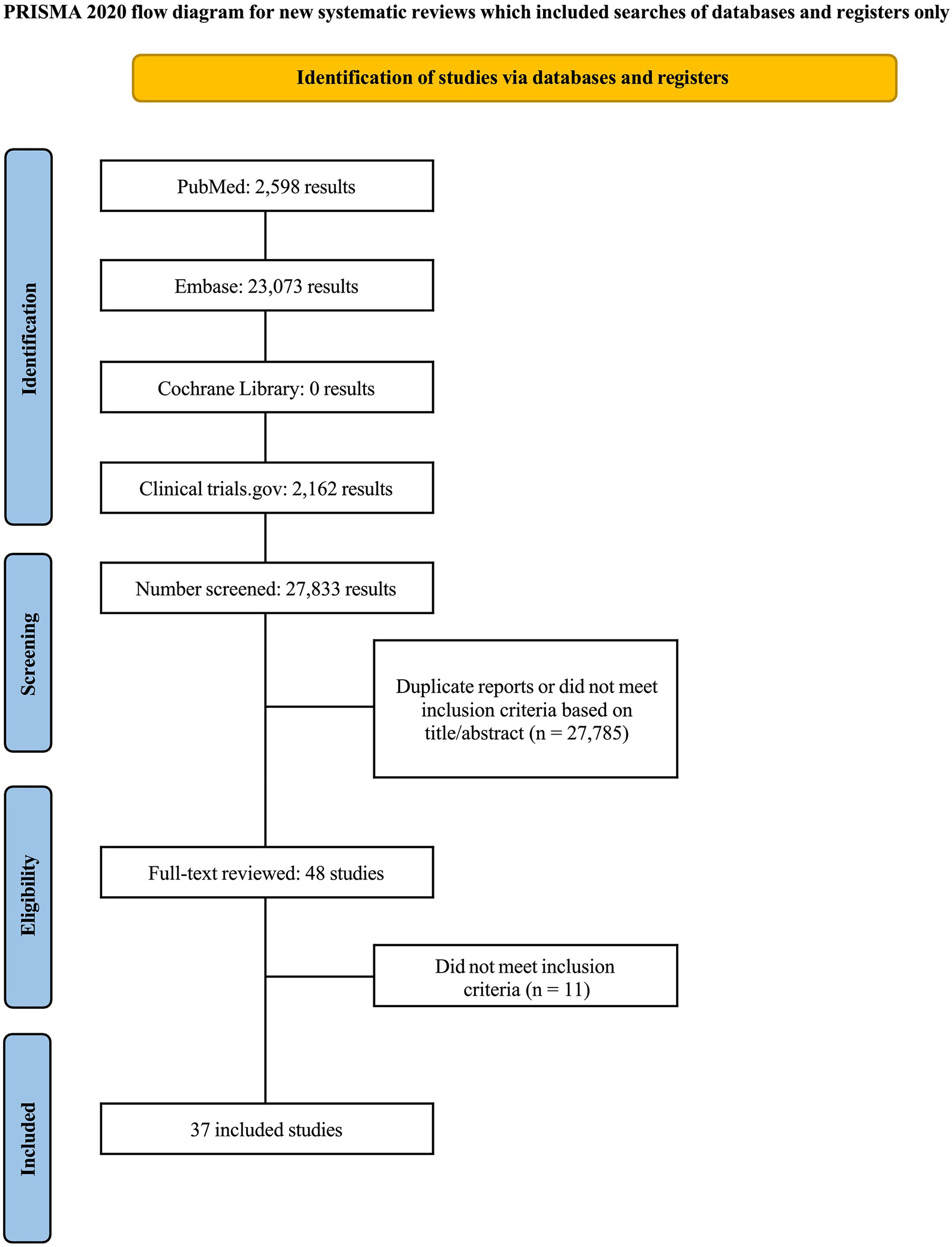
Preferred reporting items for systematic review and meta-analysis (PRISMA) flow diagram of study screening and selection.

The initial search identified 27,833 records (Fig. 1). Following the removal of duplicates and ineligible studies, 27,785 publications underwent title and abstract screening based on the predefined inclusion criteria. A total of 48 studies remained, and only 37 studies met all eligibility requirements and were included in the analysis.

Study characteristics are reported in Supplementary Table S1. A total of 11,022 patients were included in the trials. Most studies were multicenter and conducted in North America and Europe (n = 32). The risk of bias tool for randomized trials (RoB 2) was used for quality assessment. No study was considered to be at high risk of bias (Fig. S1).

### Racial and Ethnic Description

**Figure 2.**
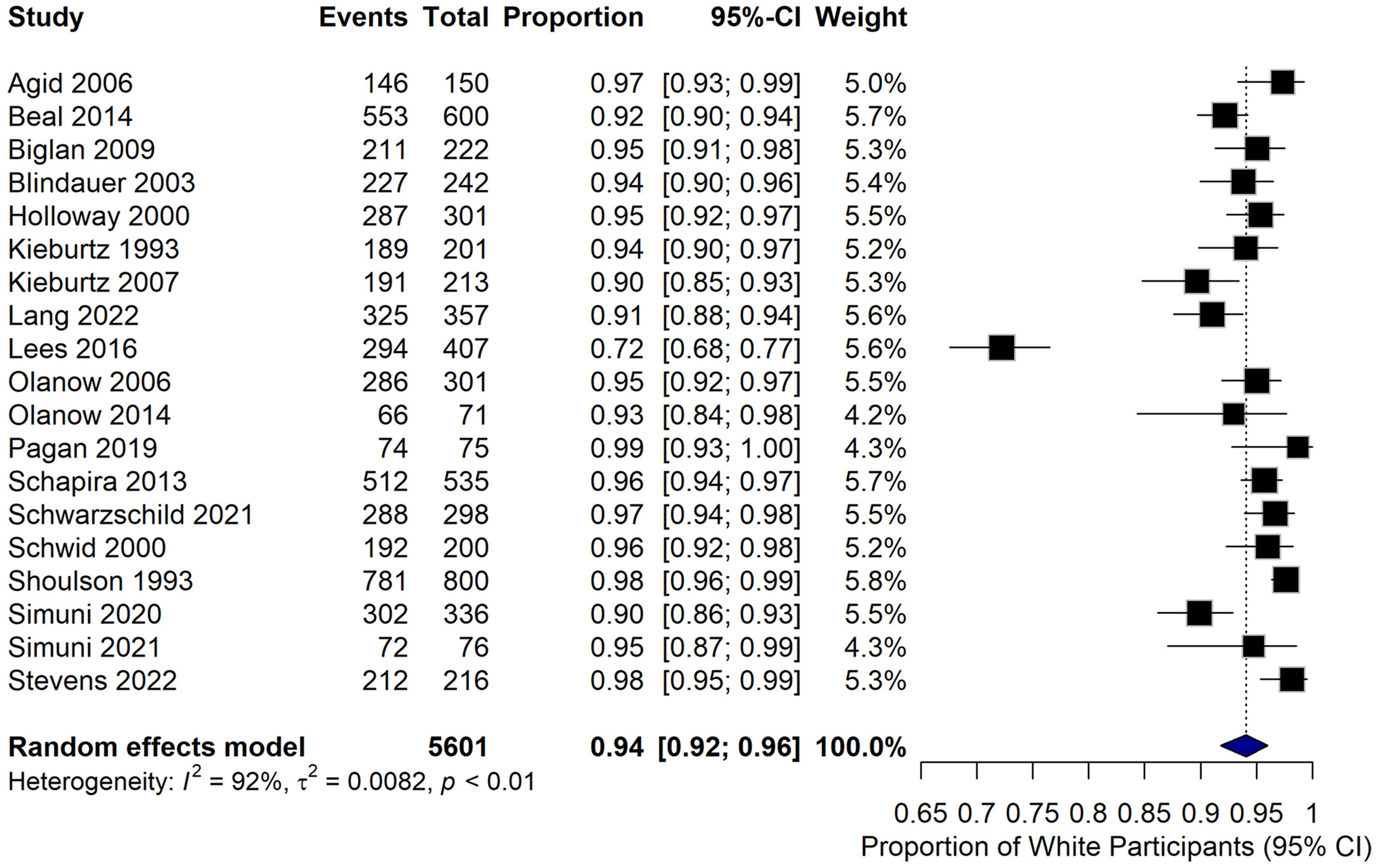
Prevalence of white participants in the clinical trials.

Of the 37 DBRCTs included in this analysis, only 19 studies (51.4%) reported race/ethnicity. From these studies, the majority of patients were white, and only 251 out of 5735 participants were categorized as non-White (4.38%). The pooled prevalence of participants identifying as White in clinical trials was 98% (CI 0.97–0.99, *I*^2^ = 95.5%, *P* < 0.0001), with 10,888 participants (Fig. 2).

Considering all included DBRCTs, the prevalence of minorities was: Asian 154/11,022 (1.4%), Black 22/11,022 (0.19%), Hispanic 19/11,022 (0.17%), Hawaiian 1/11,022 (0.009%), American Indian/Alaska 4/11,022 (0.036%), and Other/Multiracial 51/11,022 (0.46%).

When only considering studies that clearly reported race/ ethnicity in their results (N = 19), the racial and ethnic distribution of racial and ethnic minorities was: Asian 154/251 (61.4%), Black 22/251 (20.3%), Hispanic 19/251 (7.6%), Hawaiian 1/251 (0.4%), American Indian/Alaska 4/251 (1.6%) and Other/Multiracial 51/251 (20.3%) (Fig. S2).

### Sex

All DBRCTs reported sex- 63.7% were male and 36.4% female. The prevalence of being women and participating in a DBRCT was 36% (CI 0.34–0.38, *I*^2^ = 80%, *P* < 0.01) (Fig. S3).

### Other Social Determinants of Health

Educational level was rarely reported in any clinical trials (three studies). The low heterogeneity (*I*^2^ = 29%, *P* = 0.24) indicates that a high level of educational attainment was a consistent feature across these trial populations, highlighting a potential lack of diversity in socioeconomic representation (Fig. S4). The geographic setting (rural vs. urban) of the study populations was not reported in any studies.

## Discussion

This systematic review and meta-analysis evaluated 37 studies involving 11,022 patients to assess racial and ethnic representation in clinical trials of potential disease-modifying therapies for PD. To our knowledge, this is the first study evaluating the inclusion of racial and ethnic minorities in potential disease-modifying DBRCTs in PD. When evaluating all studies, only 51.4% (N = 19) reported data on racial and ethnic groups, with a disproportionately higher number of white participants. These findings underscore the lack of representativeness in most clinical trials, despite the National Institutes of Health (NIH) policy for inclusion of women and ethnic minorities in clinical trials, signed into law in 1993.^1,8^ PD is the second most common neurodegenerative disease worldwide and is projected to affect more than 12 million individuals by 2040.^9^ However, representation of individuals from racial and ethnic minorities in PD trials has been low, limiting the generalizability of results.^10,11–13^

The exclusion of people from racial and ethnic minorities in PD research reflects health inequities, as individuals from lower socioeconomic backgrounds might face compounding barriers. This includes limited access to neurological care, financial hardship, and geographic disparities in healthcare infrastructure. Such systemic obstacles might delay diagnosis, restrict treatment options, and worsen clinical outcomes. Such underrepresentation might also result in a vicious cycle, when therapies developed without diverse participation have the potential of being less effective for marginalized groups, while delayed diagnoses and inadequate treatments simultaneously increase societal burdens on caregivers and healthcare systems.^14–16,17,18,19^

Additionally, our study also highlighted the lack of the African American population in such trials. This underrepresentation is also reflected in other areas, such as in genetic research. For instance, research often favors populations with well-studied PD-associated mutations, as LRRK2 or GBA1 genes, which have been primarily investigated in individuals of European ancestry, leaving other groups underrepresented in genetic studies.^20,21,22^ Such gaps in representation fundamentally limit the generalizability of genetic findings and hinder the development of inclusive diagnostic and therapeutic frameworks. As of now, it is not clear if these specific variants might impact the disease courses or response to PD-related therapies.^23,24^ Recent evidence suggests that African American patients with PD have worse quality of life as compared to White patients, which might be related to social determinants of health, or potentially different disease trajectories.^11^

In our study, the impact of other social determinants of health in respect to the inclusion in clinical trials was also evaluated. Women were less represented in such studies, although this might be limited by the overall higher prevalence of PD in men. While educational level has been rarely reported in trials, this might have potential impacts in the disease course.^25^ For instance, several studies have recognized the impact of low educational level and cognition decline in PD.^26,27^ In our study, we have not identified any trials reporting urban or rural status of participants. Although this information might not necessarily change disease course, the rural population has been considered to have delayed diagnosis and overall lower access to clinical research in neurology. As such, they represent an important group to consider when developing efforts to improve inclusivity.^28^

To address this persistent underrepresentation, clinical trials must identify systemic and sociocultural barriers limiting diverse participation. The Fostering Inclusion in Research for Underrepresented Populations in Parkinson’s Disease (FIRE-UP PD) study has investigated key challenges in PD research, including geographic and financial constraints, distrust in medical research, and structural as lack of multilingual resources barriers. The study proposed targeted interventions, such as community-based recruitment strategies and culturally tailored outreach, to improve engagement. However, its findings were limited by the exclusion of non-English speakers and reliance on digital platforms, which may inadvertently marginalize older or technologically underserved populations.^29,30,31^

Additional strategies to increase diversity in clinical trials have been suggested.^32^ Diversifying the clinical and research team, along with financial incentives for participants to mitigate the financial burden, might be important. Additionally, educational seminars to promote self-awareness, the translation of materials into languages other than English, and adapting and validating the commonly used scales in diverse communities have been considered potential approaches to address this issue.^11,18,29^

Our study has several limitations. First, it is possible that despite being collected, racial and ethnic data were not formally reported in the paper or Supplementary Material of the identified studies. In any case, such information highlights the need for more transparency and dissemination of demographic data. Moreover, there might have been variability in how race and ethnicity were categorized, which was not consistent in all studies. While self-reporting remains the gold standard for such data collection, participants may exhibit response bias due to concerns about potential differential treatment based on disclosed demographic characteristics, potentially compromising data accuracy through non-disclosure.

This systematic review and meta-analysis highlight the low number of racial and ethnic minorities in clinical trials exploring potential disease-modifying therapies for PD. Moreover, few social determinants of health were described in detail, including educational and rural setting. This represents a significant barrier to achieving adequate representativeness, given the well-established heterogeneity in PD clinical manifestations in different populations.

Future clinical trials must prioritize the inclusion of historically marginalized populations to better characterize population-specific variations in disease risk, progression patterns, and therapeutic response. Additional research is needed to better understand this variability and promote strategies to enhance diversity in trial participation and reporting to ensure equitable therapeutic interventions across all affected populations.

## Supplementary Material

Supplementary Figure 2 **Figure S2.** Race/ethnicity representation among non-white participants, %.

Supplementary Figure 1 **Figure S1.** Risk of bias assessment in clinical trials (RoB 2).

Supplementary Figure 3 **Figure S3.** Proportion of women participants in the clinical trials.

Supplementary Figure 4 **Figure S4.** Educational years of patients.

Supplementary File 1 **Supplementary File S1.** Search methodology.

Supplementary File 2 **Supplementary File S2.** Preferred reporting items for systematic review and meta-analysis (PRISMA) checklist.

Supplementary Table 1 **Table S1.** Baseline characteristics of included studies

Supporting information may be found in the online version of this article.
