# Supplementary figures and images for: Racial and Ethnic Diversity in Clinical Trials for Disease Modifying Drugs in Parkinson Disease: A Systematic Review & Meta-Analysis
Source: Mov Disord Clin Pract. Author manuscript; Available in PMC 2026 Jan 27. (PMC12834084; doi:10.1002/mdc3.70482)

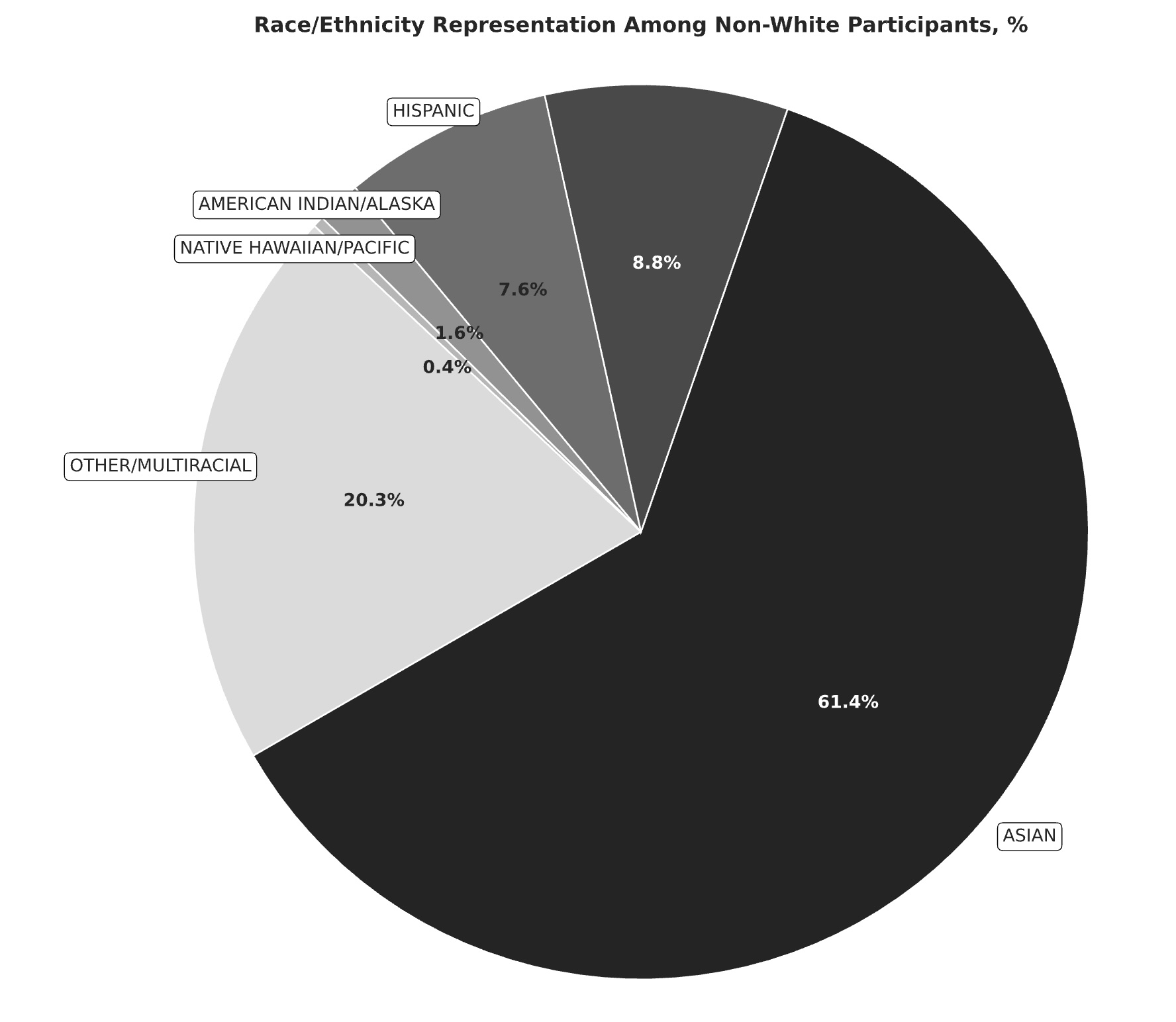

Supplement: Supplementary Figure 2 — Figure S2. Race/ethnicity representation among non-white participants, %. [file NIHMS2133623-supplement-Supplementary_Figure_2.png]

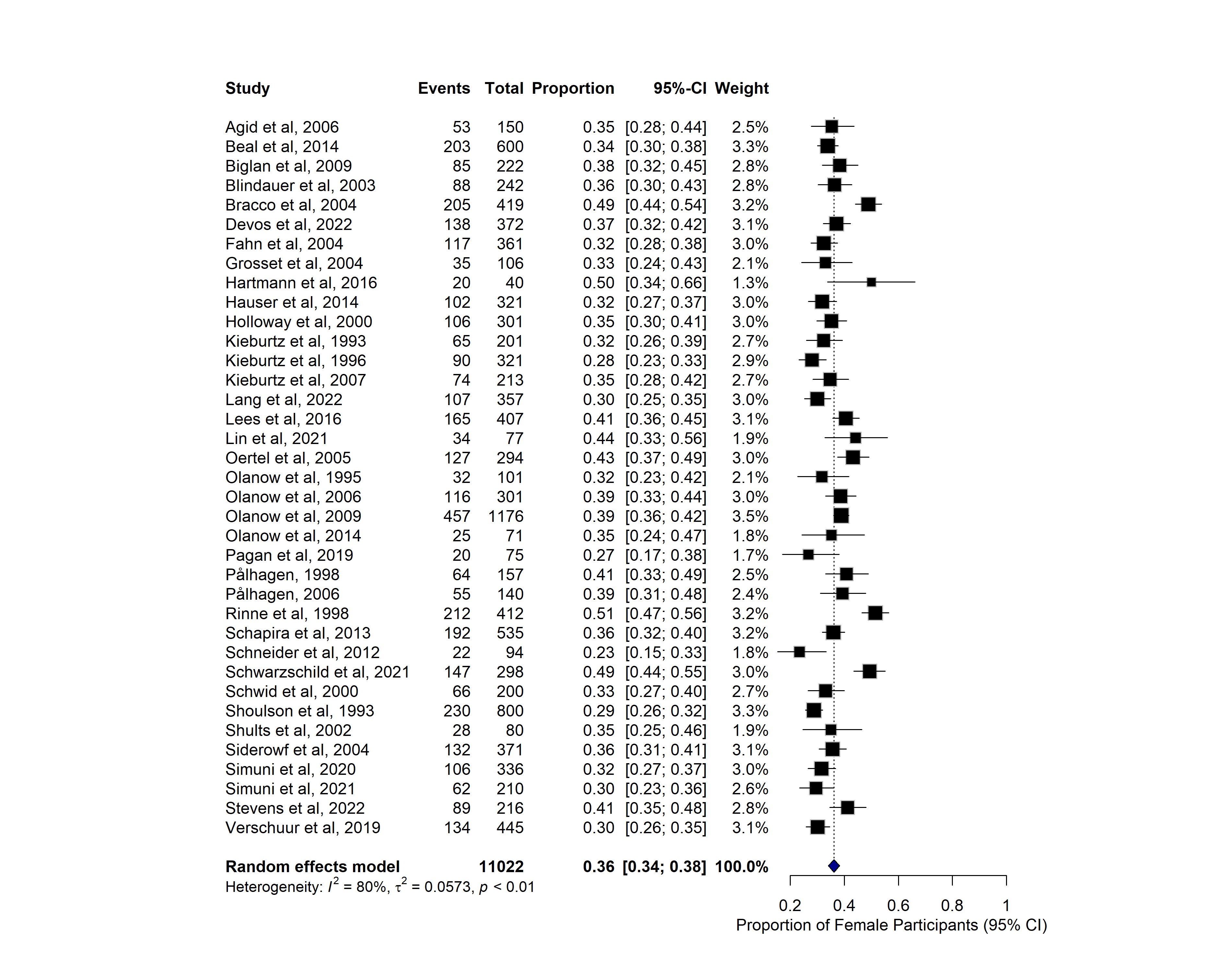

Supplement: Supplementary Figure 3 — Figure S3. Proportion of women participants in the clinical trials. [file NIHMS2133623-supplement-Supplementary_Figure_3.png]

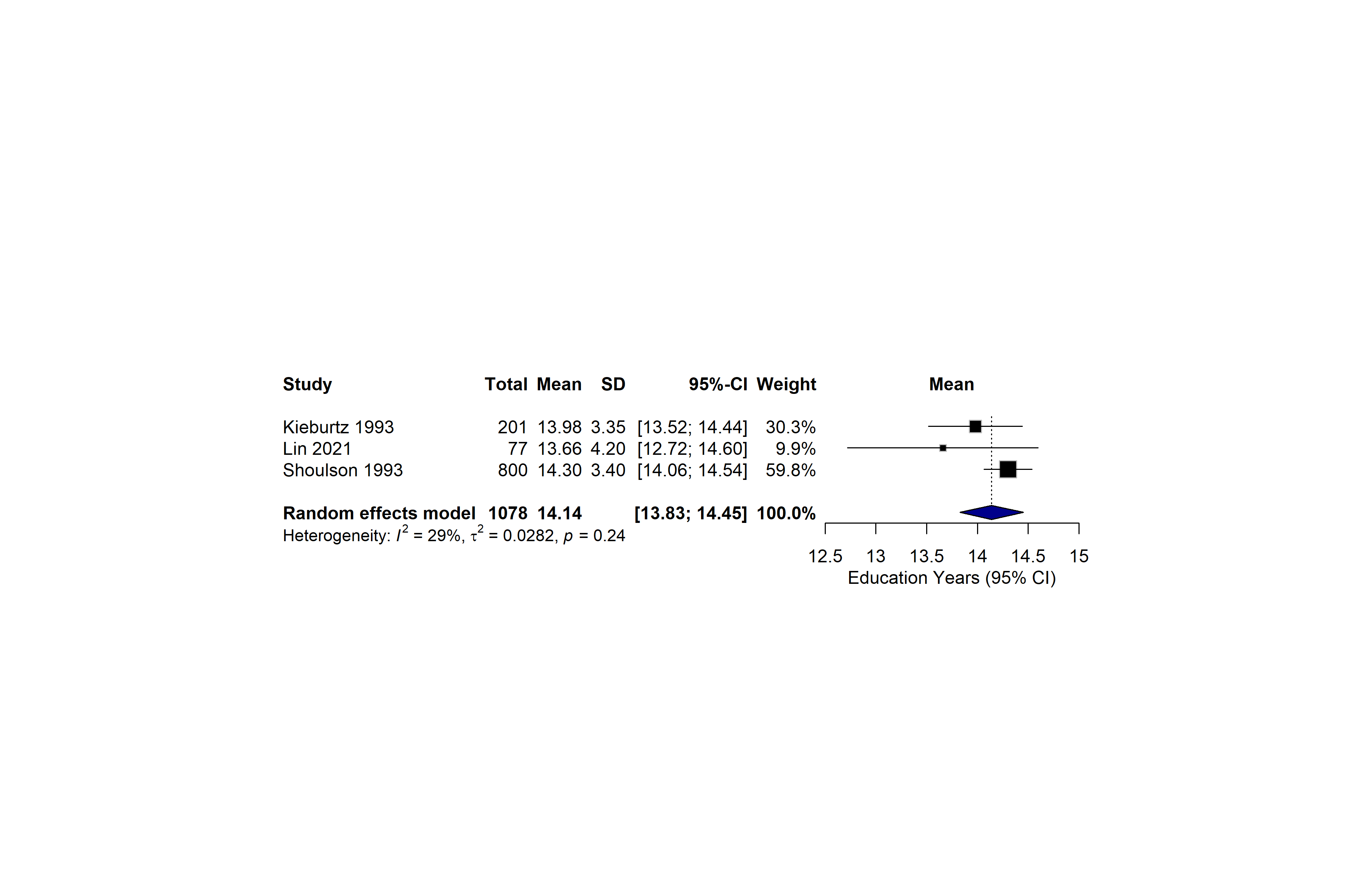

Supplement: Supplementary Figure 4 — Figure S4. Educational years of patients. [file NIHMS2133623-supplement-Supplementary_Figure_4.png]
