# Supplementary material for: Racial and Ethnic Diversity in Clinical Trials for Disease Modifying Drugs in Parkinson Disease: A Systematic Review & Meta-Analysis
Source: Mov Disord Clin Pract. Author manuscript; Available in PMC 2026 Jan 27. (PMC12834084; doi:10.1002/mdc3.70482)
Supplement: Supplementary Figure 1 — Figure S1. Risk of bias assessment in clinical trials (RoB 2). [file NIHMS2133623-supplement-Supplementary_Figure_1.pdf]

Risk of bias summary for randomized studies (RoB 2)

Study	Bias from randomization process	Bias due to deviations from intended interventions	Bias due to missing outcome data	Bias in measurement of the outcomes	Bias in selection of the reported result	Overall risk of bias
AGID 2006	Low	Low	Low	Low	Low	Low
BEAL 2014	Low	Low	Low	Low	Low	Low
BIGLAN 2009	Some concerns	Low	Low	Low	Low	Low
BLINDAUER 2003	Low	Low	Low	Low	Low	Low
BRACCO 2004	Low	Low	Low	Low	Low	Low
DEVOS 2022	Low	Low	Low	Low	Low	Low
FAHN 2004	Low	Low	Low	Low	Low	Low
GROSSET 2004	Low	Low	Low	Low	Low	Low
HARTMANN 2016	Low	Low	Low	Low	Low	Low
HAUSER 2014	Low	Low	Low	Low	Low	Low
HOLLOWAY 2000	Low	Low	Low	Low	Low	Low
KIEBURTZ 1993	Low	Low	Low	Low	Low	Low
KIEBURTZ 1996	Low	Low	Low	Low	Low	Low
KIEBURTZ 2007	Low	Low	Low	Low	Low	Low
LANG 2022	Low	Low	Low	Low	Low	Low
LEES 2016	Low	Low	Low	Low	Low	Low
LIN 2021	Low	Low	Low	Low	Low	Low
OERTEL 2005	Low	Low	Low	Low	Low	Low
OLANOW 1995	Some concerns	Low	Low	Low	Low	Low
OLANOW 2006	Low	Low	Low	Low	Low	Low
OLANOW 2009	Low	Low	Low	Low	Low	Low

OLANOW 2014	Low	Low	Low	Low	Low	Low
PAGAN 2019	Low	Low	Low	Low	Low	Low
PÅLHAGEN 1998	Low	Low	Low	Low	Low	Low
PÅLHAGEN 2006	Low	Low	Low	Low	Low	Low
RINNE 1998	Low	Low	Low	Low	Low	Low
SCHAPIRA 2013	Low	Low	Low	Low	Low	Low
SCHNEIDER 2012	Low	Low	Low	Low	Low	Low
SCHWARZSCHILD 2021	Low	Low	Low	Low	Low	Low
SCHWID 2000	Low	Low	Low	Low	Low	Low
SHOULSON 1993	Low	Low	Low	Low	Low	Low
SHULTS 2002	Low	Low	Low	Low	Low	Low
SIDEROWF 2004	Low	Low	Low	Low	Low	Low
SIMUNI 2020	Some concerns	Low	Low	Low	Low	Low
SIMUNI 2021	Some concerns	Low	Low	Low	Low	Low
STEVENS 2002	Low	Low	Low	Low	Low	Low
VERSCHUUR 2019	Low	Low	Low	Low	Low	Low

Sterne JAC, Savović J, Page MJ, Elbers RG, Blencowe NS, Boutron I, Cates CJ, Cheng H-Y, Corbett MS, Eldridge SM, Hernán MA, Hopewell S, Hróbjartsson A, Junqueira DR, Jüni P, Kirkham JJ, Lasserson T, Li T, McAleenan A, Reeves BC, Shepperd S, Shrier I, Stewart LA, Tilling K, White IR, Whiting PF, Higgins JPT. RoB 2: a revised tool for assessing risk of bias in randomised trials. *BMJ* 2019; **366**: l4898.
