# Supplementary material for: Racial and Ethnic Diversity in Clinical Trials for Disease Modifying Drugs in Parkinson Disease: A Systematic Review & Meta-Analysis
Source: Mov Disord Clin Pract. Author manuscript; Available in PMC 2026 Jan 27. (PMC12834084; doi:10.1002/mdc3.70482)
Supplement: Supplementary File 1 — Supplementary File S1. Search methodology. [file NIHMS2133623-supplement-Supplementary_File_1.pdf]

Methods:

A medical librarian (DT) searched the literature for records including the major concepts of Parkinson disease and disease-modifying clinical trials. Search strategies combined the use of standardized vocabulary (MeSH and Emtree) and keywords in PubMed, and Embase databases. All searches were limited to clinical trial studies on humans and in English. Clinicaltrials.gov was searched for all completed trials (n=2162). A total of 25,671 results were uploaded into EndNote and 1,449 duplicates were removed for a new total of 24,222. Full reproducible search strategies provided below.

PubMed
Searched conducted: March 7, 2023
Results: 2598

Search Strategy: (("early Parkinson disease"[tiab:~3] OR "early stage Parkinson"[tiab:~3] OR "Parkinson disease" [Mesh] OR "Parkinsons"[ti] OR "parkinson disease"[tiab:~1] OR "Parkinson Disease"/pc OR "Parkinson Disease"[Mesh])) AND (("disease modifying drug"[tiab:~1] OR "disease modifying drugs"[tiab:~1] OR "disease modifying therapy"[tiab:~1] OR "disease modifying therapies"[tiab:~1] OR "disease modifying treatment"[tiab:~1] OR "disease modification"[tiab:~1] OR "disease modifying interventions"[tiab:~1] OR "early treatment"[tiab:~1] OR "slowing disease progression"[tiab:~1] OR "slower progression"[tiab:~1] OR "neuroprotection agent" [tiab:~1] OR "neuroprotective agent"[tiab:~1] OR "Neuroprotective Agents"[Mesh] OR "neuroprotective treatment"[tiab:~1] OR "progression"[tiab] OR "progression disability"[tiab:~2] OR "slower progression"[tiab:~1] OR "delay disability"[tiab:~1] OR "progression of disability"[tiab:~1] OR "Disease Progression"/pc OR "Disease Progression"[Mesh] OR "Antiparkinson Agents"[Mesh]) OR ("Double-blind method"[Mesh])) AND Filters: Clinical Trial, Clinical Trial Protocol, Clinical Trial, Phase I, Clinical Trial, Phase II, Clinical Trial, Phase III, Clinical Trial, Phase IV, Comparative Study, Controlled Clinical Trial, Randomized Controlled Trial, English, Humans

Embase
Search Conducted: March 11, 2023
Results: 23,073

Search Strategy: (‘early Parkinson disease’):ti OR (‘early stage Parkinson’):ti OR (‘Parkinson Disease’/exp) OR (‘Parkinsons’) AND (‘disease modifying’):ti OR (‘disease modifying drug’):ti OR (‘disease modifying drugs’):ti OR (‘disease modifying therapy’):ti OR (‘disease modifying therapies’):ti OR (‘disease modifying treatment’):ti OR (‘disease modification’):ti OR (‘disease modifying interventions’):ti OR (‘early treatment’):ti OR (‘slowing disease progression’):ti OR (‘slower progression’):ti OR (‘neuroprotection agent’):ti OR (‘neuroprotective agent’):ti OR (‘Neuroprotective Agents’/exp) OR (‘neuroprotective treatment’):ti OR (‘progression’):ti OR (‘slower progression’):ti OR (‘delay disability’):ti OR (‘progression disability’):ti OR (‘Disease Progression’/exp) OR (‘Antiparkinson Agents’/exp) AND ('clinical trial'/de OR 'comparative effectiveness'/de OR 'comparative study'/de OR 'controlled clinical trial'/de OR 'controlled study'/de OR 'double blind procedure'/de OR 'human'/de OR 'major clinical study'/de OR 'multicenter study'/de OR 'phase 1 clinical trial'/de OR 'phase 2 clinical trial'/de OR 'phase 3 clinical trial'/de OR 'randomized controlled trial'/de)

Limited to English language

N=23,073

Cochrane Library
Searched conducted March 7, 2024

(“early Parkinson disease” OR “early stage Parkinson” OR Parkinson disease OR “Parkinson’s” OR “parkinson disease” OR “Parkinson Disease” OR “Double-Blind Method”) AND ("disease modifying drug" OR "disease modifying drugs" OR "disease modifying therapy" OR “disease modifying therapies” OR "disease modifying treatment" OR "disease modification" OR “disease modifying interventions" OR “early treatment” OR “slowing disease progression" OR “slower progression” OR "neuroprotection agent" OR "neuroprotective agent" OR “Neuroprotective Agents” OR "neuroprotective

treatment" OR "progression" OR “progression disability” OR “slower progression” OR “delay disability” OR “progression of disability” OR “Disease Progression” OR “Antiparkinson Agents”)

No reviews found

Clinical trials.gov
Search conducted: March 7, 2024
Completed trials: 2,162

Search Strategy: ("disease modifying drug" OR "disease modifying drugs" OR "disease modifying therapy" OR “disease modifying therapies” OR "disease modifying treatment" OR "disease modification" OR “disease modifying interventions" OR “early treatment” OR “slowing disease progression" OR “slower progression” OR "neuroprotection agent" OR "neuroprotective agent" OR “Neuroprotective Agents” OR "neuroprotective treatment" OR "progression" OR “progression disability” OR “slower progression” OR “delay disability” OR “progression of disability” OR “Disease Progression” OR “Antiparkinson Agents”) AND (“early Parkinson disease” OR “early stage Parkinson” OR Parkinson disease OR “Parkinson’s” OR “parkinson disease” OR “Parkinson Disease” OR “Double-Blind Method”)
