# Supplementary material for: Racial and Ethnic Diversity in Clinical Trials for Disease Modifying Drugs in Parkinson Disease: A Systematic Review & Meta-Analysis
Source: Mov Disord Clin Pract. Author manuscript; Available in PMC 2026 Jan 27. (PMC12834084; doi:10.1002/mdc3.70482)
Supplement: Supplementary File 2 — Supplementary File S2. Preferred reporting items for systematic review and meta-analysis (PRISMA) checklist. [file NIHMS2133623-supplement-Supplementary_File_2.pdf]

**PRISMA 2020 Checklist (Updated)**

Manuscript Title: Racial and Ethnic Diversity in Clinical Trials for Disease Modifying Drugs in Parkinson Disease: A Systematic Review & Meta-Analysis

| Section | Checklist Item | Location in Manuscript |
|---|---|---|
| Title | Identify the report as a systematic review and meta-analysis. | Page 1, Title |
| Abstract | Structured abstract with background, objectives, methods, results, and conclusions. | Abstract, Page 1 |
| Rationale | Describe the rationale for the review in the context of existing knowledge. | Introduction, Lines 40–60 |
| Objectives | Provide an explicit statement of the objective(s). | Introduction, Lines 61–68 |
| Eligibility Criteria | Specify inclusion and exclusion criteria for the review. | Methods, Lines 70–78 |
| Information Sources | Describe all information sources (databases, registers, etc.). | Methods, Lines 79–84 |
| Search Strategy | Present the full search strategy for at least one database. | Supplementary File 1 |
| Selection Process | Specify the methods used to select studies. | Methods, Lines 80–88 |
| Data Collection Process | Describe the method of data extraction. | Methods, Lines 88–94 |
| Data Items | List and define all variables for which data were sought. | Methods, Lines 90–100 |

| | | |
|---|---|---|
| Study Risk of Bias Assessment | Describe the methods used to assess risk of bias. | Methods, Lines 100–108; Supplementary Figure 1 |
| Effect Measures | Specify for each outcome the effect measure(s) used. | Methods, Lines 108–112 |
| Synthesis Methods | Describe the methods used to synthesize results. | Methods, Lines 112–118 |
| Reporting Bias Assessment | Describe any methods used to assess reporting bias. | Not applicable; all included studies assessed for bias via RoB 2. |
| Certainty Assessment | Describe any methods used to assess certainty of evidence. | Not applicable; descriptive synthesis only. |
| Results – Study Selection | Give numbers of studies screened, assessed, and included, with reasons for exclusions. | Results, Lines 120–135; Figure 1 (PRISMA flow diagram) |
| Results – Study Characteristics | Describe characteristics of included studies. | Results, Lines 135–150; Supplementary Table 1 |
| Results – Risk of Bias in Studies | Present risk of bias assessments. | Results, Lines 150–160; Supplementary Figure 1 |
| Results – Individual Study Results | Present results for each study. | Results, Lines 160–175 |
| Results – Synthesis of Results | Present results of syntheses (meta-analysis). | Results, Lines 175–185; Figure 2 |
| Discussion – Summary of Evidence | Summarize main findings including strength of evidence. | Discussion, Lines 190–205 |
| Discussion – Limitations | Discuss limitations of the evidence and review processes. | Discussion, Lines 205–220 |
| Discussion – Conclusions | Provide interpretation and implications for practice and research. | Discussion, Lines 220–235 |

| | | |
|---|---|---|
| Funding | Describe sources of funding and role of funders. | Disclosures, Page 12 |
| Conflicts of Interest | Declare any potential conflicts of interest. | Disclosures, Page 12 |
