# Supplementary material for: Racial and Ethnic Diversity in Clinical Trials for Disease Modifying Drugs in Parkinson Disease: A Systematic Review & Meta-Analysis
Source: Mov Disord Clin Pract. Author manuscript; Available in PMC 2026 Jan 27. (PMC12834084; doi:10.1002/mdc3.70482)
Supplement: Supplementary Table 1 — Table S1. Baseline characteristics of included studies [file NIHMS2133623-supplement-Supplementary_Table_1.pdf]

Supplementary Table 1 – Baseline characteristics of included studies.

Author, Year	Country/Region	Drug	Males, %
Agid, 2006	North America, Europe	Tolcapone	64,6
Beal, 2014	USA	Coenzyme Q10	66,1
Biglan, 2009	North America	Pramipexole	61,7
Blindauer, 2003	Multicenter[a]	Rotigotine	63,6
Bracco, 2004	Europe and Latin America	Cabergoline	51,1
Devos, 2022	Europe	Deferiprone	62,9
Fahn, 2004	North America	Carbidopa-Levodopa	67,6
Grosset, 2004	UK	Pergolide	66,9
Hartmann, 2016	France	Bee venom	50
Hauser, 2014	USA	Rasagiline	68,2
Holloway, 2000	North America	Pramipexole	64,7
Kieburtz, 1993	USA	Lazabemide	67,6
Kieburtz, 1996	USA	Lazabemide	71,9
Kieburtz, 2007	USA	Coenzyme Q10 and GPI-1485	65,2
Lang, 2022	North America, Europa	Cinpanemab	70
Lees, 2016	Africa, North and South Americas, Asia, Oceania	Opicapone and Levodopa	59,4
Lin, 2021	Taiwan	Lovastatin	55,8
Oertel, 2005	Europe and South Africa	Pergolide	56,8
Olanow, 1995	USA	Deprenyl	68,3
Olanow, 2006	Africa, North and South Americas, Asia, Oceania	TCH346	61,4
Olanow, 2009	Africa, North and South Americas, Asia, Oceania	Rasagiline	61,1
Olanow, 2014	Germany, USA and New Zealand	Levodopa-Carbidopa Intestinal Gel	64,7
Pagan, 2019	USA	Nilotinib	73,3
Pålhagen, 1998	Sweden	Selegiline	59,2
Pålhagen, 2006	Sweden	Selegiline	60,7
Rinne, 1998	Europe, USA and Oceania	Cabergoline	48,5
Schapira, 2013	USA	Pramipexole	64,1
Schneider, 2012	USA	Ganglioside GM1	76,6
Schwarzschild, 2021	USA	Inosine	50,6
Schwid, 2000	USA	Remacemide	67
Shoulson, 1993	USA	Tocopherol and Deprenyl	71,3
Shults, 2002	USA	Coenzyme Q10	65
Siderowf, 2004	North America	Rasagiline	64,4
Simuni, 2020	North America	Isradipine	68,5
Simuni, 2021	North America	Poliglitazone	70,5
Stevens, 2022	UK	Simvastatin	58,8
Verschuur, 2019	Netherlands	Levodopa	69,9

[a] *Countries were not specified*
